# A single preoperative FGF23 measurement is a strong predictor of outcome in patients undergoing elective cardiac surgery: a prospective observational study

**DOI:** 10.1186/s13054-015-0925-6

**Published:** 2015-04-23

**Authors:** Timo Speer, Heinrich V Groesdonk, Beate Zapf, Vanessa Buescher, Miriam Beyse, Laura Duerr, Stella Gewert, Patrizia Krauss, Aaron Poppleton, Stefan Wagenpfeil, Danilo Fliser, Hans-Joachim Schaefers, Matthias Klingele

**Affiliations:** Department of Internal Medicine, Nephrology and Hypertension, Saarland University Medical Centre, Kirrberger Strasse, D-66424 Homburg/Saar, Germany; Department of Thoracic and Cardiovascular Surgery, Saarland University Medical Centre, Homburg/Saar, Germany; Department of Anaesthesiology, Intensive Care and Pain Therapy, Saarland University Medical Centre, Homburg/Saar, Germany; Institute for Medical Biometry, Epidemiology and applied Medical Informatics, Saarland University Medical Centre, Homburg/Saar, Germany

## Abstract

**Introduction:**

Several scoring systems have been developed to predict postoperative mortality and complications in patients undergoing cardiac surgery. However, these computer-based calculations are time- and cost-intensive. A simple but highly predictive test for postoperative risk would be of clinical benefit with respect to increasingly scarce hospital resources. We therefore assessed the predictive power of fibroblast growth factor 23 (FGF23) measurement compared with an established scoring system.

**Methods:**

We conducted a prospective interdisciplinary observational study at the Saarland University Medical Centre that included 859 patients undergoing elective cardiac surgery between January 2010 and March 2011 with a median follow-up after discharge of 822 days. We compared a single preoperative measurement of FGF23 as a prognostic tool with the 18 parameters comprising EuroSCORE II with respect to postoperative mortality, acute kidney injury, non-occlusive mesenteric ischemia, clinical course and long-term outcome.

**Results:**

Preoperative FGF23 levels were highly predictive of postoperative outcome and complications. The predictive value of FGF23 for mortality in the receiver operating characteristic curve was greater than the EuroSCORE II (area under the curve: 0.800 versus 0.725). Moreover, preoperative FGF23 independently predicted postoperative acute kidney injury and non-occlusive mesenteric ischemia comparably to the EuroSCORE II. Finally, FGF23 was found to be an independent predictor of clinical course parameters, including duration of surgery, ventilation time and length of stay.

**Conclusions:**

In patients undergoing elective cardiac surgery, a simple preoperative FGF23 measurement is a powerful indicator of surgical mortality, postoperative complications and long-term outcome. Its utility compares to the widely used EuroSCORE II.

**Electronic supplementary material:**

The online version of this article (doi:10.1186/s13054-015-0925-6) contains supplementary material, which is available to authorized users.

## Introduction

An increasingly elderly population has resulted in greater cardiovascular disease prevalence and an associated need for cardiac surgery [1,2]. Despite several studies demonstrating improved survival and quality of life data in elderly patients undergoing cardiac surgery, non-elective surgery is associated with a two to three times greater risk of death [3-5]. Even though risk of perioperative morbidity is greater in these patients, age does not represent an absolute contraindication for surgical intervention, with patient selection remaining the ultimate predictor of clinical outcome [5]. In order to assess the risk of patient mortality prior to cardiac surgery, scoring systems have been developed that incorporate risk factors such as age [6,7]. The EuroSCORE II is a widely used and well-established scoring system comprising 18 distinct variables. However, to date, simple preoperative prognostic biomarkers have remained unavailable.

Fibroblast growth factor 23 (FGF23) has recently emerged as a powerful biomarker for adverse outcome in patients with or without chronic kidney disease (CKD) [8,9]. The hormone FGF23 is secreted by osteoblasts [10], resulting in increased phosphate excretion [11] and decreased renal 1α-hydroxylation of 25-OH vitamin D [12,13]. FGF23 levels begin to rise early within the course of CKD [14]. Although the precise physiological mechanism remains uncertain, phosphate accumulation due to impaired renal function is thought to induce secretion of FGF23, thereby increasing renal phosphate excretion and maintaining serum phosphate levels within a physiological range. In spite of this, elevated FGF23 levels are associated with worse clinical outcomes in patients with CKD [15-17]. Interestingly, an association between FGF23 levels, impaired left ventricular (LV) function and atrial fibrillation has been reported in patients with CKD and even in patients without renal failure [18,19]. Furthermore, FGF23 predicts risk of cardiovascular mortality and progression to congestive cardiac failure in patients with stable coronary artery disease [9].

The effect of FGF23 on its physiological target organs (that is, the kidneys and the parathyroid glands) depends on its co-receptor α-Klotho. The *Klotho* gene has a role within the aging process, and different FGF-Klotho co-receptors exist within the human body [20].

In the present study, we evaluated the predictive power of a preoperative FGF23 measurement as a biomarker of postoperative complications, clinical course and in-hospital mortality, as well as of long-term outcome after hospital discharge, in patients undergoing elective cardiac surgery.

## Materials and methods

### Study design and setting

From January 2010 to March 2011, all patients scheduled for elective cardiac surgery within the Department of Thoracic and Cardiovascular Surgery at the Saarland University Medical Centre in Germany were screened for inclusion. Exclusion criteria included age <18 years, refusal to participate, emergency admissions for cardiac surgery, haemodynamic instability necessitating emergency cardiac surgery, or inability to give written consent for participation. The study was designed as a prospective cohort study and was approved by the local ethics committee (Landesärztekammer des Saarlandes reference ID 199/09). Written informed consent was obtained from all patients included in this study.

Clinical data were obtained during an initial patient interview and subsequent review of medical documentation. Patient demographic and perioperative data were entered into a databank. Patients were characterised according to smoking status with respect to calculated total pack-year history. Patients with current use of antidiabetic medications, repeated blood glucose levels >250 mg/dl or haemoglobin A1c levels >7% were categorised as having diabetes mellitus. Arterial angiography was performed in patients with suspected non-occlusive mesenteric ischaemia (NOMI) after the surgical procedure [21], with the diagnosis made according to the vascular scoring system described by Minko *et al*. [22]. Postoperative acute kidney injury (AKI) was defined on the basis of Acute Kidney Injury Network (AKIN) criteria [23] (that is, an AKIN score ≥1).

### Observation period after discharge

Each participant had a follow-up telephone interview between October 2012 and December 2012, corresponding to 2 to 3 years after hospital discharge. The following data were collected: date of death (if applicable), cause of death, myocardial infarction, angina pectoris with consecutive hospital admission, percutaneous transluminal coronary angioplasty and/or stenting, coronary artery bypass graft, resuscitation, hospitalization due to heart failure, stroke, initiation of renal replacement therapy, New York Heart Association stage and intake of diuretics. In case of mortality, a family member was contacted. When neither the patient nor a relative could be contacted, the general practitioner, cardiologist or nephrologist to whom a letter of discharge had been sent was contacted. In cases where this was unsuccessful, the registry office was questioned if the patient had died.

### Laboratory parameters and measurements

Blood samples for biochemical monitoring were processed in the central laboratory of the Saarland University Medical Centre. Blood samples were obtained under standard conditions alongside routine preoperative laboratory assessment within 24 hours prior to surgery and centrifuged at 2,800 × *g* for 10 minutes at 4°C. Supernatants were stored in aliquots at −80°C. C-terminal FGF23 levels were measured from plasma samples by enzyme-linked immunosorbent assay (lowest cutoff value of 3 relative units (RU)/ml, highest cutoff value of 2,000 RU/ml; Immutopics International, San Clemente, CA, USA). The results are reported as relative units, whereby 1 RU/ml equates to 2 pg/ml according to the manufacturer’s guidelines.

Patients were categorised into tertiles according to FGF23 level, allowing assessment of risk for the endpoints death, kidney failure, NOMI and long-term mortality through comparison of three clinically simple categories: low, medium and high risk.

### EuroSCORE II

To predict early mortality after cardiac surgery, a scoring system was developed based on a panel of risk factors [7]. As mortality associated with cardiac surgery has decreased significantly over the last 15 years, the former EuroSCORE I has been superseded by EuroSCORE II, which incorporates 18 factors proven to influence risk [6]. All patients were preoperatively categorised according to their EuroSCORE II.

### Statistical analysis

Continuous variables are expressed as mean ± standard deviation (normally distributed variables) or as median and interquartile range (IQR) (skewed variables). Categorical variables are presented as a percentage unless otherwise stated. For comparisons between continuous variables, the Kruskal-Wallis *H* test was used. Categorical variables were compared by use of the χ^2^ test or Fisher’s exact test. The association between continuous variables was assessed by Spearman’s rank correlation testing.

To examine the association between FGF23 and AKI as well as NOMI during hospitalisation, binary logistic regression analyses were performed, including FGF23 divided into tertiles as a categorical variable. Moreover, additional models were calculated, adjusting for potential confounders (age, sex, mean arterial blood pressure, sinus rhythm, coronary artery disease, chronic heart failure, diabetes, serum creatinine and serum high-sensitivity C-reactive protein (hsCRP)). An additional model was built, adjusting for EuroSCORE II, comprising 18 variables as described above. Furthermore, linear regression analyses were used to determine the association between log-transformed FGF23 as a continuous variable and different metric variables during hospitalisation (duration of surgery and cardiopulmonary bypass, time of hypothermic circulatory arrest, ventilation time, length of stay on the intensive care unit (ICU), and length of in-hospital stay). Finally, to assess the effect of increasing serum FGF23 level on mortality during follow-up, Cox regression analyses were performed using FGF23 stratified into tertiles and adjusted for age, sex, mean arterial blood pressure, sinus rhythm, coronary artery disease, chronic heart failure, diabetes, serum creatinine and serum hsCRP. Similar calculations were performed for the N-terminal fragment of the prohormone B-type brain natriuretic peptide (NT-proBNP) divided into tertiles or using log-transformed NT-proBNP. Additionally, Kaplan-Meier survival plots were used to examine the relationship between tertiles of FGF23 and mortality after discharge. For binary logistic regression analyses, c-statistics are reported.

To compare the power of FGF23 and EuroSCORE II in predicting mortality and the occurrence of AKI and NOMI, receiver operating characteristic (ROC) curve analyses were performed, with the area under the curve (AUC) reported for each parameter. Statistical differences between the AUC under two different ROC curves were examined by using the Hanley-McNeil test.

Two-sided *P*-values <0.05 were considered statistically significant. All statistical analyses were carried out using SPSS 20.0 software (IBM, Armonk, NY, USA).

## Results

### Participants and descriptive data

A total of 1,163 patients underwent elective cardiac surgery with extracorporeal circulation within our centre during the screening period. Among these, 865 patients met the study inclusion criteria. Six patients were subsequently excluded from analysis for lack of blood samples for measurement of FGF23. The remaining 859 patients were divided into 3 groups according to preoperative FGF23 level. The patients’ baseline characteristics are presented in Table 1. The patients’ mean age (with standard deviation (SD)) was 63.7 ± 1.46 years, and 68.6% of participants were male. The patients in the highest FGF23 tertile were more likely to be older, female or have preexisting diabetes. Indications for cardiac surgery and LV ejection fraction were comparable between tertiles. However, median pro-BNP was six times higher in patients in the highest FGF23 tertile than among those in the lowest tertile. Moreover, patients with endocarditis and patients requiring resternotomy had significantly higher FGF23 serum concentrations (data not shown).

**Table 1:** **Baseline characteristics of study participants** ^**a**^

**Variable**	**FGF23**			**All, n = 859**	***P*** **-value** ^**b**^
	**Tertile 1 (≤50.6 RU/ml), n = 287**	**Tertile 2 (50.7 to 89.9 RU/ml), n = 286**	**Tertile 3 (≥90.0 RU/ml), n = 286**		
Male sex (%)	80.8	69.9	54.9	68.6	<0.001
Age (yr)	59.1 ± 14.9	63.7 ± 14.3	68.3 ± 13.2	63.7 ± 1.46	<0.001
Indication for cardiac surgery^c^					
Valvulopathy (%)	72.5	69.2	68.7	70.1	0.561
Coronary artery disease (%)	34.5	40.9	37.7	37.7	0.285
Aortic disease (%)	35.2	28.0	23.2	28.8	0.006
Mean arterial blood pressure (mmHg)	94 ± 30	90 ± 36	82 ± 38	89 ± 35	<0.001
Sinus rhythm (%)	92.3	84.6	76.8	84.6	<0.001
Diabetes mellitus (%)	14.6	26.1	35.4	24.8	<0.001
Coronary artery disease (%)	36.2	45.5	47.2	42.9	0.018
Chronic cardiac failure (%)	90.2	85.7	83.1	86.3	0.042
Ejection fraction (%)	50 ± 27	49 ± 25	48 ± 26	49 ± 26	0.542
EuroSCORE II	2.6 (1.4 to 5.2)	3.7 (2.0 to 6.8)	5.7 (2.7 to 12.4)	3.8 (1.9 to 7.4)	<0.001
FGF23 (RU/ml)	39.2 (31.7 to 45.4)	64.5 (56.5 to 75.3)	182.9 (114.5 to 416.5)	64.5 (45.4 to 114.5)	<0.001
Serum creatinine (mg/dl)	0.98 ± 0.20	1.06 ± 0.26	1.53 ± 1.35	1.19 ± 0.84	<0.001
Serum NT-proBNP (pg/ml)	212 (90 to 612)	572 (170 to 1,415)	1,276 (443 to 3,575)	525 (156 to 1,507)	<0.001
Serum hsCRP (mg/L)	1.6 (0.7 to 4.5)	2.4 (0.9 to 6.1)	5.2 (2.1 to 16.3)	2.6 (1.0 to 8.1)	<0.001
Duration of surgery (min)	156 ± 43	171 ± 55	185 ± 67	171 ± 57	<0.001
Duration of cardiopulmonary bypass (min)	75 ± 32	80 ± 36	92 ± 48	82 ± 40	<0.001
Time of hypothermic circulatory arrest (min)	51 ± 22	51 ± 24	58 ± 34	53 ± 27	0.001
Ventilation time (hr)	9 (8 to 13)	11 (8 to 16)	14 (10 to 29)	11 (8 to 18)	<0.001
Length of ICU stay (days)	1 (1 to 1)	1 (1 to 1)	1 (1 to 3)	1 (1 to 2)	<0.001
Total length of stay (days)	9 (8 to 11)	10 (9 to 12)	11 (9 to 16)	10 (8 to 13)	<0.001
Acute kidney injury (%)	11.8	23.1	45.8	26.8	<0.001
Non-occlusive mesenteric ischaemia (%)	3.8	6.6	16.5	9.0	<0.001
Death during hospitalisation (%)	0.0	2.1	6.7	2.9	<0.001
Death during follow-up (%)	3.1	5.6	12.2	7.0	<0.001

^a^FGF23, Fibroblast growth factor 23; hsCRP, High-sensitivity C-reactive protein; ICU, Intensive care unit; NT-proBNP, N-terminal fragment of the prohormone B-type brain natriuretic peptide; RU, Relative units. Values are presented as mean ± standard deviation, median (interquartile range) or percentage where appropriate. ^b^ *P*-value for the comparison between groups according to tertiles of FGF23. ^c^Multiple indications are possible.

### Preoperative FGF23 level as predictor of postoperative mortality

**Figure 1:**
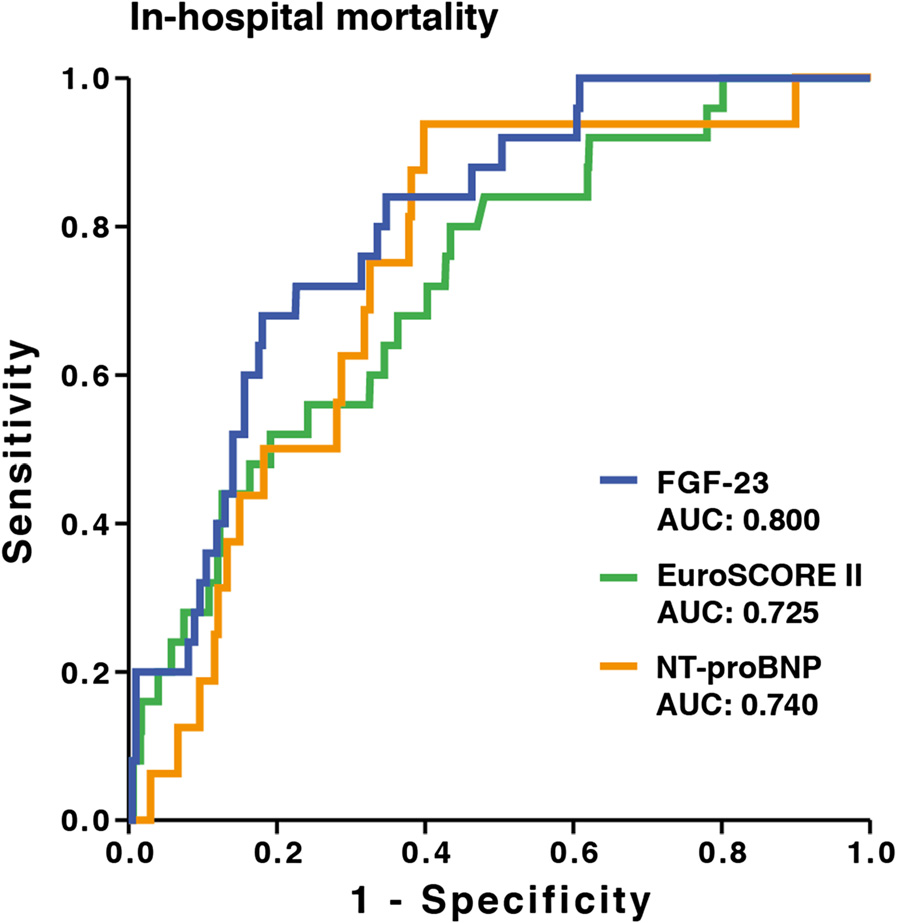
Receiver operating characteristic curve analyses of the prediction of in-hospital mortality. AUC, Area under the curve; FGF23, Fibroblast growth factor 23; NT-proBNP, N-terminal fragment of the prohormone B-type brain natriuretic peptide FGF23.

Total in-hospital mortality was 2.9% (that is, half that predicted by EuroSCORE II). FGF23No inpatient mortality was noted in the lowest FGF23 tertile, compared with 2.1% and 6.7% mortality in the other two tertiles. According to EuroSCORE II, predictive mortality was 4.0%, 6.0% and 8.0% in the lowest to highest tertiles, respectively. ROC curve analysis for FGF23, EuroSCORE II and NT-proBNP for prediction of surgical mortality is presented in Figure 1. The predictive value of preoperative FGF23 level was comparable to that of the EuroSCORE II (AUC: 0.800 versus 0.725; *P* = 0.172) and even higher than NT-proBNP (AUC: 0.740).

### Preoperative FGF23 level as predictor of outcome after hospital discharge

**Figure 2:**
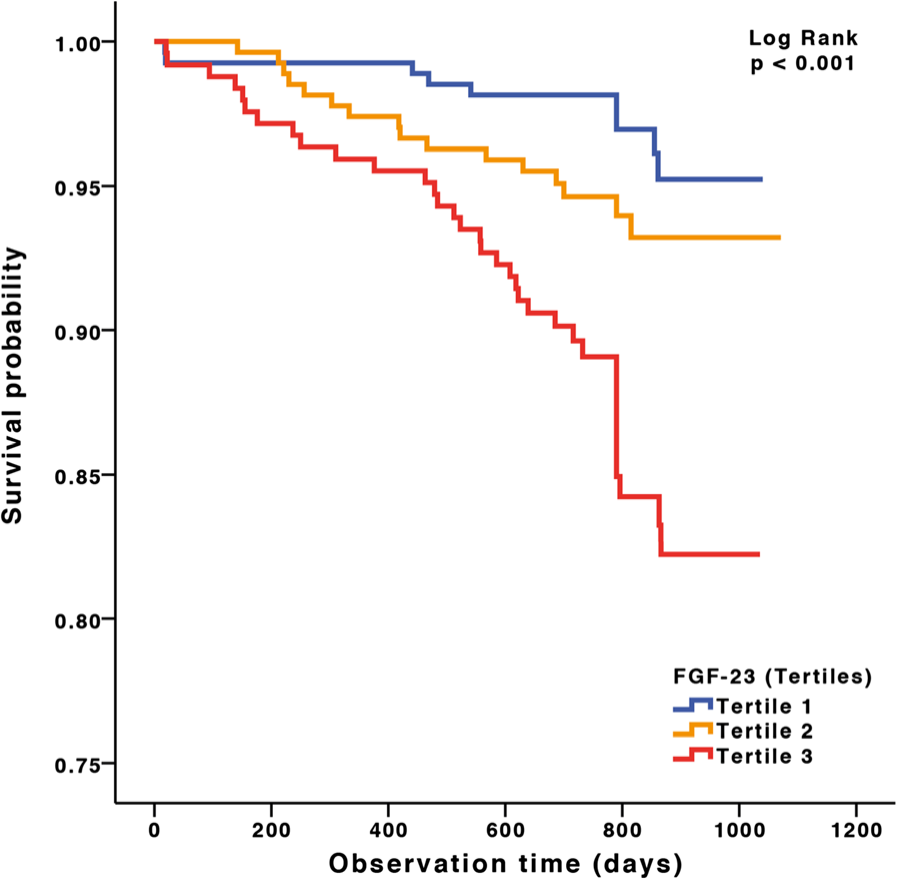
Kaplan-Meier survival plots for tertiles of fibroblast growth factor 23 during long-term follow-up. FGF23, Fibroblast growth factor 23.

A significant association was noted between preoperative FGF23 level and mortality during the long-term observation period after hospital discharge (Figure 2). During a median follow-up period of 822 (IQR: 359 to 1,071) days, mortality was 4.47 times higher in patients in the highest FGF23 tertile than in the lowest tertile (Table 2). Even after adjustment for age, sex, mean arterial blood pressure, sinus rhythm, coronary artery disease, chronic heart failure, diabetes, and serum creatinine and hsCRP levels, mortality remained 2.34 times higher in patients within the highest FGF23 tertile than in the lowest tertile (Table 2), even after adjustment for potential confounders or EuroSCORE II. In contrast, NT-proBNP did not significantly predict mortality after hospital discharge (Additional file 1).

**Table 2:** **Cox and logistic regression analyses for FGF23 tertiles and mortality during follow-up, postoperative acute kidney injury and non-occlusive mesenteric ischaemia** ^**a**^

**Model**	**FGF23**	**Mortality**		**AKI** ^**b**^		**NOMI** ^**c**^	
		**HR (95% CI)**	***P*** **-value**	**HR (95% CI)**	***P*** **-value**	**HR (95% CI)**	***P*** **-value**
Crude	Tertile 1	1	–	1	–	1	–
	Tertile 2	1.86 (0.82 to 4.21)	0.137	2.23 (1.42 to 3.51)	<0.001	1.83 (0.85 to 3.92)	0.121
	Tertile 3	4.40 (2.11 to 9.18)	<0.001	6.26 (4.08 to 9.59)	<0.001	4.79 (2.42 to 9.48)	<0.001
Adjusted model 1^d^	Tertile 1	1	–	1	–	1	–
	Tertile 2	1.46 (0.64 to 3.36)	0.370	1.94 (1.21 to 3.10)	0.006	1.53 (0.71 to 3.32)	0.281
	Tertile 3	3.05 (1.42 to 6.55)	0.004	4.89 (3.09 to 7.72)	<0.001	3.44 (1.69 to 7.02)	0.001
Adjusted model 2^e^	Tertile 1	1	–	1	–	1	–
	Tertile 2	1.35 (0.59 to 3.11)	0.482	1.65 (1.01 to 2.67)	0.044	1.24 (0.56 to 2.75)	0.590
	Tertile 3	2.34 (1.07 to 5.29)	0.034	2.69 (1.62 to 4.47)	<0.001	2.76 (1.30 to 5.85)	0.008
Adjusted model 3^f^	Tertile 1	1	–	1	–	1	–
	Tertile 2	1.88 (0.83 to 4.25)	0.131	1.96 (1.24 to 3.10)	0.004	1.59 (0.74 to 3.42)	0.239
	Tertile 3	4.54 (2.18 to 9.45)	<0.001	4.67 (3.00 to 7.27)	<0.001	3.71 (1.84 to 7.48)	<0.001

^a^AKI, Acute kidney injury; CI, Confidence interval; FGF23, Fibroblast growth factor 23; HR, Hazard ratio; NOMI, Non-occlusive mesenteric ischaemia; RU, Relative units. FGF23 tertile 1: ≤50.6 RU/ml, tertile 2: 50.7 to 89.9 RU/ml, tertile 3: ≥90 RU/ml. ^b^c-Statistics for the prediction of AKI in adjusted model 3: 0.690. ^c^c-Statistics for the prediction of NOMI in adjusted model 3: 0.590. ^d^Adjusted for age and sex. ^e^Adjusted for age, sex, mean arterial blood pressure, sinus rhythm, coronary artery disease, chronic heart failure, diabetes, serum creatinine and serum high-sensitivity C-reactive protein. ^f^Adjusted for EuroSCORE II, comprising 18 variables as described in the Methods section.

### Preoperative FGF23 level as predictor of postoperative complications and clinical course

The incidence of postoperative complications such as AKI differed significantly between patients in different FGF23 tertiles. Patients in the highest FGF23 tertile had a six times higher likelihood of developing AKI after cardiac surgery compared with those in the lowest tertile. Moreover, preoperative FGF23 level was a significant and independent predictor of occurrence of postoperative AKI (Table 2).

The likelihood of NOMI after cardiac surgery was 4.8 times higher in patients in the highest FGF23 tertile compared with patients in the lowest tertile. Even after adjustment for confounding variables, preoperative FGF23 level remained an independent risk factor for NOMI (Table 2).

In similar models, patients in the highest tertile of NT-proBNP had a significantly elevated risk for postoperative AKI (*P* = 0.001). However, we could not reveal a significant association between NT-proBNP and the occurrence of NOMI (Additional file 1).

The predictive value of FGF23 and EuroSCORE II for AKI was comparable, with AUC values of 0.715 and 0.719 (*P* = 0.892), respectively (Additional file 2). Interestingly, even the predictive value of FGF23 and EuroSCORE II for NOMI was comparable (AUC values of 0.692 and 0.702, respectively; *P* = 0.838).

The preoperative FGF23 level was independently associated with duration of surgical procedure and postoperative cardiorespiratory support, as well as with parameters of overall clinical course, such as ventilation time, length of stay on the ICU and length of stay in the hospital (Table 3 and Additional file 3). Contrarily, NT-proBNP was not significantly associated with time of hypothermic circulatory arrest, ventilation time or ICU length of stay.

**Table 3:** **Linear regression analyses for tertiles of FGF23 and N-terminal fragment of the prohormone B-type brain natriuretic peptide with different outcome measures** ^**a**^

		**FGF23**		**NT-proBNP**	
**Dependent variable**	**Tertile**	**β (95% CI)**	***P*** **-value**	**β (95% CI)**	***P*** **-value**
Duration of surgery	Tertile 1	Reference			
	Tertile 2	12.5 (3.5 to 21.6)	0.007	11.2 (0.6 to 21.7)	0.038
	Tertile 3	20.7 (10.6 to 30.8)	<0.001	27.9 (16.0 to 39.9)	<0.001
Duration of cardiopulmonary bypass	Tertile 1	Reference			
	Tertile 2	6.5 (−0.2 to 12.9)	0.057	4.6 (−2.7 to 12.0)	0.215
	Tertile 3	16.2 (8.9 to 23.4)	<0.001	22.1 (13.8 to 30.4)	<0.001
Time of hypothermic circulatory arrest	Tertile 1	Reference			
	Tertile 2	0.4 (−0.5 to 1.2)	0.441	−0.7 (−1.8 to 0.4)	0.241
	Tertile 3	2.0 (1.0 to 3.0)	<0.001	0.3 (−0.9 to 1.6)	0.600
Ventilation time	Tertile 1	Reference			
	Tertile 2	2.3 (−12.3 to 16.8)	0.761	−9.6 (−22.7 to 3.5)	0.149
	Tertile 3	24.8 (8.6 to 40.9)	0.003	6.6 (−8.2-21.3)	0.384
Length of ICU stay	Tertile 1	Reference			
	Tertile 2	0.2 (−0.5 to 0.9)	0.550	−0.5 (−1.2 to 0.2)	0.166
	Tertile 3	1.3 (0.5 to 2.1)	0.001	0.3 (−0.5 to 1.1)	0.444
Total length of stay	Tertile 1	Reference			
	Tertile 2	0.6 (−0.7 to 1.8)	0.365	0.9 (−0.3 to 2.1)	0.133
	Tertile 3	1.8 (0.4 to 3.1)	0.009	2.0 (0.6 to 3.3)	0.005

^a^CI, Confidence interval; FGF23, Fibroblast growth factor 23; ICU, Intensive care unit; NT-proBNP, N-terminal fragment of the prohormone B-type brain natriuretic peptide; RU, Relative units. Data are adjusted for age, sex, mean arterial blood pressure, sinus rhythm, coronary artery disease, chronic heart failure, diabetes, serum creatinine and serum high-sensitivity C-reactive protein. Other models for FGF23 are shown in Additional file 3. For FGF23, tertile 1: ≤50.6 RU/ml, tertile 2: 50.7 to 89.9 RU/ml, tertile 3: ≥90 RU/ml; for NT-proBNP, tertile 1: ≤222 pg/ml, tertile 2: 223 to 1,058 pg/ml, tertile 3: ≥1,059 pg/ml.

### Preoperative measurement of FGF23 and patient risk assessment

**Figure 3:**
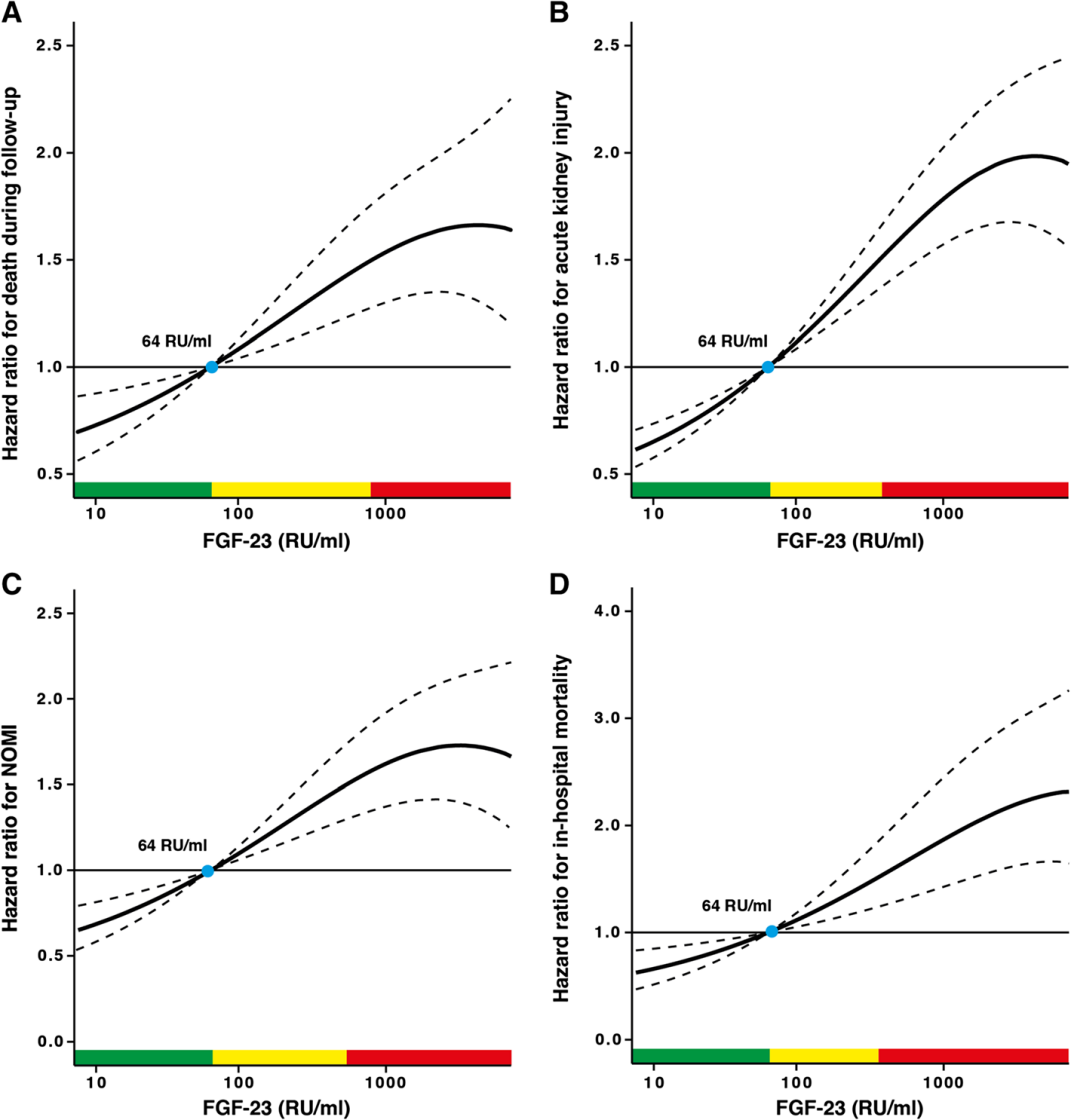
Plot of multivariable adjusted hazard ratios according to preoperative levels of fibroblast growth factor 23. **(A)** Mortality during follow-up. **(B)** Acute kidney injury. **(C)** Non-occlusive mesenteric ischaemia (NOMI). **(D)** In-hospital mortality. Solid lines represent the hazard ratios (HRs), and dashed lines the respective 95% confidence intervals. The median of fibroblast growth factor 23 (FGF23) (64 relative units (RU)/ml) was chosen as the reference value (HR =1). Data are adjusted for age, sex, mean arterial blood pressure, sinus rhythm, coronary artery disease, chronic heart failure, diabetes, serum creatinine and serum high-sensitivity C-reactive protein. Green areas represent the range of FGF23 with a HR <1, yellow areas with HR ≥1 and <1.5 and red areas with HR ≥1.5.

Because FGF23 levels predict short- and long-term postoperative outcome in patients undergoing elective cardiac surgery, a single FGF23 measurement could be helpful in individual preoperative risk assessment. For this purpose, we analysed multivariable adjusted hazard ratios for the endpoints mortality, AKI, NOMI and long-term outcome according to preoperative FGF23 level (Figure 3A to D). The individual relative risk for acute mortality, AKI, NOMI or mortality during follow-up according to the individual preoperative FGF23 level can be easily depicted by comparison with the median preoperative FGF23 level of 64 RU/ml (hazard ratio = 1.0).

## Discussion

In this large, prospective cohort study, we demonstrate the strong predictive power of a single preoperative FGF23 measurement for in-hospital complications and mortality, as well as for long-term outcome, in patients undergoing elective cardiac surgery.

### FGF23 and postoperative mortality

Preoperative serum FGF23 serum level predicts postoperative mortality at least as well as EuroSCORE II. The EuroSCORE I was developed in 1999 to predict early mortality in patients undergoing cardiac surgery based on patient, cardiological and operation-related factors [7]. Because the mortality associated with cardiac surgery has decreased significantly over the past 15 years, EuroSCORE II was introduced, incorporating additional factors proven to influence risk of mortality [6]. Despite such important modifications, the predictive capacity of EuroSCORE II for mortality was not superior to a single preoperative FGF23 measurement. Thus, on the basis of our present study, we report the novel finding of preoperative FGF23 level as a strong predictor of mortality in patients undergoing cardiac surgery.

### FGF23 as a long-term predictive marker

We found a clear association between FGF23 levels and mortality during the follow-up period. This finding confirms former studies, including the ‘Heart and Soul Study’, which demonstrated an association between FGF23 and outcomes in non-CKD patients with prevalent coronary heart disease [24], and the Uppsala Longitudinal Study of Adult Men, in which FGF23 was independently associated with elevated cardiovascular mortality during a median follow-up time of 9.7 years [25].

Although the relationship between FGF23, increased mortality and cardiovascular disease is well established in both CKD and non-CKD populations [16,18,24,26,27], the underlying pathophysiological mechanisms remain poorly understood. This association of FGF23 and increased mortality and cardiovascular disease was previously described as being independent of renal function and increased phosphate intake [26], as well as of traditional risk factors for cardiovascular events (age, obesity, hypertension, smoking and dyslipidaemia) [24,26,28,29]. Taken together, FGF23 has an undeniable capacity to predict long-term outcome; however, it remains unclear whether this is independent of factors linked to cardiovascular disease risk or renal function.

### FGF23 and acute kidney injury

AKI is a common complication after cardiac surgery [30-32], with a prevalence of up to 30%. FGF23 is a well-established biomarker for progression in patients with diabetic and non-diabetic CKD [15,33]. However, FGF23 has only recently been proposed as a potential predictor of AKI irrespective of preexisting CKD [34]. Our results clearly reveal preoperative FGF23 level as a strong and independent predictor of AKI in a large cohort of patients undergoing elective cardiac surgery.

### FGF23 and non-occlusive mesenteric ischaemia

NOMI is a rare but serious complication diagnosed by angiography after cardiac surgery [22,35,36]. Altered intestinal microcirculation could result from surgical intervention, postoperative cardiorespiratory support, vasopressor use or potentially the manifestation of a separate disease process [37-39]. During the observation period, 9% of the patients in our study population developed NOMI. The relatively high incidence may be explained by the high number of patients with advanced cardiac failure [21,40] and by the fact that clinically suspected NOMI was confirmed by intestinal angiography [21,22].

Preoperative FGF23 level was associated with an increased risk of developing NOMI. Importantly, FGF23 remained an independent predictor of NOMI even after statistical adjustment for known risk factors in the development of postsurgical mesenteric ischaemia. Because increased FGF23 levels have been linked to impaired endothelium-dependent vasodilatation in CKD [41], a similar pathophysiological mechanism could account for the association between FGF23 and NOMI. Furthermore, FGF23 has been linked to the process of vascular calcification [20,42], and these patients could be at higher risk of developing NOMI than patients without vascular calcification. When these factors are taken together, FGF23 may be deemed a potent predictive marker for vascular vulnerability in different organs.

### FGF23 and cardiac function

Although high levels of FGF23 seem to be associated with impaired LV function [18,19,43], preexisting cardiac failure and preoperative cardiac ejection fraction did not differ between FGF23 tertiles. Furthermore, regression analyses adjusted for mean arterial blood pressure, cardiac rhythm, coronary artery disease and congestive cardiac failure did not significantly impact the prognostic power of FGF23. This disagreement with previous findings [18,19,43] may be accounted for by differences in study populations. Cardiac function with regard to LV ejection fraction or preexisting cardiac failure was generally lower in our population compared with data reported by Seiler and colleagues [19]. Accordingly, at baseline, both serum NT-proBNP levels (525 pg/ml versus 220 pg/ml, respectively) and median plasma FGF23 (64.5 RU/ml versus 38.6 RU/ml, respectively) were higher in our cohort compared with the cohort studied by Seiler *et al*. Although we were unable to confirm an association between FGF23 and impaired cardiac function, we observed a significant association between FGF23 and atrial fibrillation, as Seiler *et al*. recently reported [19].

Faul and colleagues revealed a Klotho-independent causal role for FGF23 in the pathogenesis of LV hypertrophy [43]. Seiler and colleagues noted a highly significant and independent association between LV hypertrophy and function within their study population [19]. However, in patients with X-linked hypophosphatemia, a rare disease of primary FGF23 excess [44], data on cardiac structure are inconsistent with respect to the development of LV hypertrophy [43,45]. Further studies are required to clarify any potential association between FGF23 and LV function independent of LV hypertrophy.

### FGF23 as predictive marker beyond cardiac and renal function

Previous studies have shown increased FGF23 levels to be independently associated with mortality in patients with CKD [17] and patients undergoing dialysis [16]. Moreover, several studies have shown FGF23 to be a significant predictor of cardiovascular outcome independent of abnormalities of renal function [19,24,33] or mineral metabolism [46]. Interestingly, our data suggest that this predictive power is not limited to cardiac or renal disease. Preoperative FGF23 levels were an independent predictor of duration of surgery, duration of cardiorespiratory support and the time of hypothermic circulatory arrest. It is possible that by predicting duration of surgery and time of hypothermic circulatory arrest, FGF23 may indirectly predict duration of postoperative cardiorespiratory support. Even after adjusting for preexisting cardiac or renal dysfunction, FGF23 remained a significant and independent predictor of duration of surgery, duration of cardiorespiratory support and the time of hypothermic circulatory arrest. Furthermore, FGF23 independently predicted clinical course parameters such as ventilation time, duration of intensive care and total hospital stay. This may at least in part be explained by its predictive capacity for severe postoperative complications such as AKI and NOMI. Notably, FGF23 outperformed NT-proBNP as a predictor of in-hospital mortality, occurrence of AKI and NOMI and mortality postdischarge, as well as of clinical course parameters, underscoring the applicability of FGF23 as a general predictor for risk associated with cardiac surgery. Moreover, the costs of measuring FGF23 are comparable to those required to measure NT-proBNP.

### Limitations

Despite a numerically large study cohort, the majority of participants were of Caucasian ancestry, with data obtained within a single tertiary medical centre. Further multicentre studies are necessary to corroborate the present findings and to assess their significance and applicability within a primary care setting. The data presented are representative only of patients admitted for elective cardiac surgery. Patients requiring emergency cardiac surgery were excluded for practical reasons to avoid ethical issues (that is, informed consent) by *a priori* study design. Further studies are necessary to evaluate whether these findings could be expanded to patients admitted for emergency cardiac surgery.

## Conclusions

A single measurement of serum FGF23 is a powerful prognostic tool to predict complications, mortality and clinical course in patients undergoing cardiac surgery. Because patient selection is the primary means of positively influencing clinical outcome, especially in elderly patients [5], serum FGF23 may aid such clinical selection. In comparison to the well-established EuroSCORE II calculation comprising 18 distinct variables, a single measurement of serum FGF23 is equally effective in predicting outcome and allows for convenient and simple risk assessment. Moreover, FGF23 measurements could offer the possibility for screening risk prior to admission within a primary care setting, offering major potential cost benefits.

## Key messages

- Fibroblast growth factor 23 (FGF23) is a hormone which increases renal phosphate excretion. Elevated levels of FGF23 were described primarily in the context of impaired renal function and as being associated with worse clinical outcomes in patients with cardiac and/or chronic kidney disease.
- Preoperatively determined serum levels of FGF23 seem helpful in assessing the risk for postoperative complications, including acute renal failure and mortality, in patients undergoing elective cardiac surgery.
- In patients undergoing elective cardiac surgery, a simple preoperative FGF23 measurement is a powerful indicator of surgical mortality, postoperative complications, and long-term outcome. Its utility compares well with the widely used EuroSCORE II comprising 18 distinct variables, and it outperforms NT-proBNP.

## Additional files

Additional file 1: Table S1. Cox and logistic regression analyses for NT-proBNP tertiles and mortality during follow-up, postoperative acute kidney injury (AKI) and non-occlusive mesenteric ischaemia (NOMI). Additional file 2: Figure S1. ROC analyses for FGF23 and EuroSCORE II for the prediction of (a) acute kidney injury and (b) non-occlusive mesenteric ischaemia. Additional file 3: Table S2. Logistic regression analyses for FGF23 tertiles and surgery-related complications.

## Abbreviations

AKI: Acute kidney injury
AUC: Area under the curve
CI: Confidence interval
CKD: Chronic kidney disease
FGF23: Fibroblast growth factor 23
HR: Hazard ratio
hsCRP: High-sensitivity C-reactive protein
ICU: Intensive care unit
LV: Left ventricular
NOMI: Non-occlusive mesenteric ischaemia
NT-proBNP: N-terminal fragment of the prohormone B-type brain natriuretic peptide
ROC: Receiver operating characteristic
RU: Relative unitsFGF23
