# Supplementary material for: A single preoperative FGF23 measurement is a strong predictor of outcome in patients undergoing elective cardiac surgery: a prospective observational study
Source: Crit Care. 2015 Apr 23;19(1):190. doi: 10.1186/s13054-015-0925-6 (PMC4424828; doi:10.1186/s13054-015-0925-6)
Supplement: Additional file 1: Table S1. — Cox and logistic regression analyses for NT-proBNP tertiles and mortality during follow-up, postoperative acute kidney injury (AKI) and non-occlusive mesenteric ischaemia (NOMI). [file 13054_2015_925_MOESM1_ESM.pdf]

Supplemental Table 1: Cox and logistic regression analyses for NT-proBNP tertiles and mortality during follow-up, post-operative acute kidney injury (AKI) and non-occlusive mesenteric ischaemia (NOMI)

Model	NT-proBNP	Mortality		AKI[1]		NOMI[2]	
		HR (95% CI)	*P*	HR (95% CI)	*P*	HR (95% CI)	*P*
**Crude**	Tertile 1	1	…	1	…	1	…
	Tertile 2	1.44 (0.46-4.54)	0.532	2.66 (1.40-5.04)	0.003	1.48 (0.41-5.34)	0.546
	Tertile 3	6.07 (2.34-15.72)	<0.001	8.36 (4.56-15.36)	<0.001	6.46 (2.17-19.22)	0.001
**Adjusted model 1***	Tertile 1	1	…	1	…	1	…
	Tertile 2	0.88 (0.27-2.88)	0.834	1.80 (0.92-1.80)	0.088	1.05 (0.28-3.97)	0.938
	Tertile 3	3.09 (1.10-8.69)	0.032	5.11 (2.65-9.83)	<0.001	4.06 (1.25-13.19)	0.020
**Adjusted model 2[§]**	Tertile 1	1	…	1	…	1	…
	Tertile 2	0.88 (0.27-2.89)	0.835	1.59 (0.80-3.17)	0.191	0.94 (0.25-3.63)	0.933
	Tertile 3	2.63 (0.89-7.75)	0.079	3.53 (1.75-7.13)	<0.001	3.51 (1.02-12.09)	0.046
**Adjusted model 3[#]**	Tertile 1	1	…	1	…	1	…
	Tertile 2	0.85 (0.26-2.78)	0.786	1.55 (0.78-3.11)	0.213	0.89 (0.23-3.45)	0.860
	Tertile 3	2.13 (0.70-6.46)	0.182	3.31 (1.63-6.73)	0.001	2.95 (0.84-10.41)	0.093

NT-proBNP tertile 1: ≤222 pg/ml, tertile 2: 223-1058 pg/ml, tertile 3: ≥1059 pg/ml

* adjusted for age and gender

[§] adjusted for age, gender, mean arterial blood pressure, sinus rhythm, coronary artery disease, chronic heart failure, diabetes, serum-creatinine, serum high-sensitive C-reactive protein (CRP)

[#] adjusted for EuroSCORE II comprising 18 variables, as described in the methods section

[1] c-statistics for the prediction of AKI in adjusted model 3: 0.646

[2] c-statistics for the prediction of NOMI in adjusted model 3: 0.500
