# Supplementary figures and images for: A single preoperative FGF23 measurement is a strong predictor of outcome in patients undergoing elective cardiac surgery: a prospective observational study
Source: Crit Care. 2015 Apr 23;19(1):190. doi: 10.1186/s13054-015-0925-6 (PMC4424828; doi:10.1186/s13054-015-0925-6)

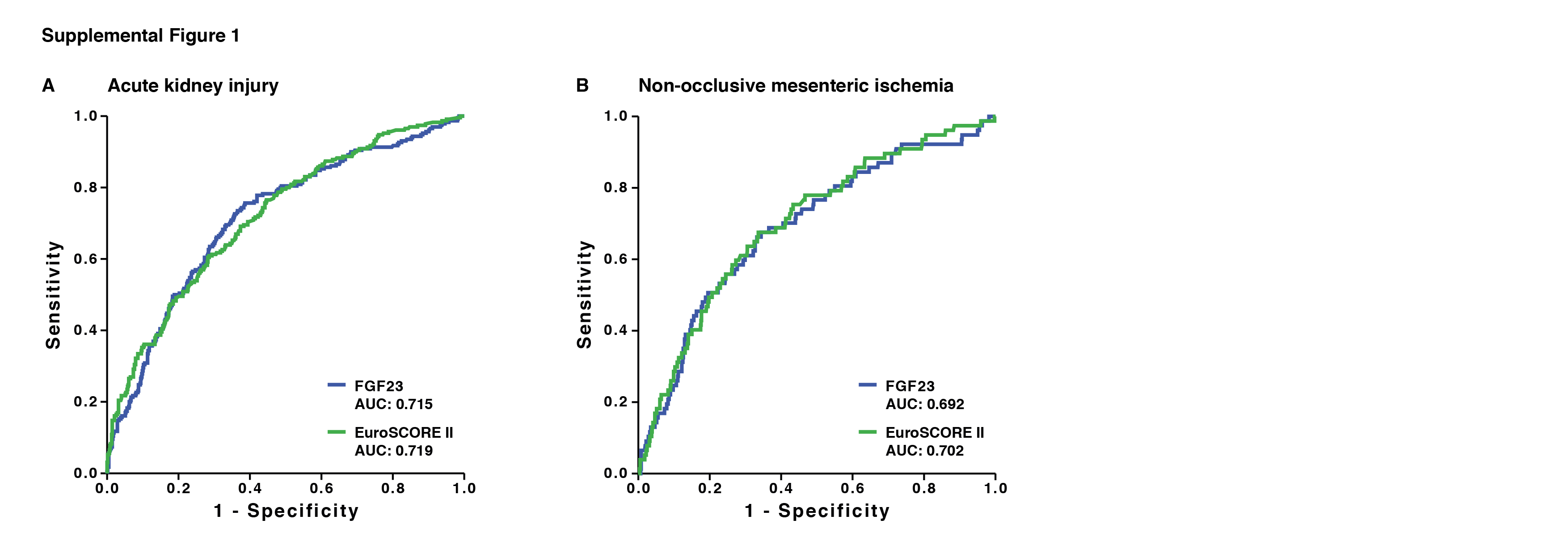

Supplement: Additional file 2: Figure S1. — ROC analyses for FGF23 and EuroSCORE II for the prediction of (a) acute kidney injury and (b) non-occlusive mesenteric ischaemia. [file 13054_2015_925_MOESM2_ESM.png]
