# Supplementary material for: A single preoperative FGF23 measurement is a strong predictor of outcome in patients undergoing elective cardiac surgery: a prospective observational study
Source: Crit Care. 2015 Apr 23;19(1):190. doi: 10.1186/s13054-015-0925-6 (PMC4424828; doi:10.1186/s13054-015-0925-6)
Supplement: Additional file 3: Table S2. — Logistic regression analyses for FGF23 tertiles and surgery-related complications. [file 13054_2015_925_MOESM3_ESM.docx]

**Supplemental Table 2**

| **Dependent Variable** | **Tertile of FGF23** | **Crude** | | **Adjusted 1*** | | **Adjusted 2^§^** | | **Adjusted 3^#^** | |
| --- | --- | --- | --- | --- | --- | --- | --- | --- | --- |
|  |  | **B (95 % CI)** | ***P*** | **B (95 % CI)** | ***P*** | **B (95 % CI)** | ***P*** | **B (95 % CI)** | ***P*** |
| **Duration of surgery** | 1 | Reference | | | | | | | |
|  | 2 | 15.1 (6.0–24.2) | 0.001 | 15.1 (6.0–24.4) | 0.001 | 12.8 (3.6–22.0) | 0.007 | 10.4 (1.7–19.2) | 0.020 |
|  | 3 | 28.9 (19.7–38.0) | <0.001 | 29.1 (19.5–38.8) | <0.001 | 25.8 (16.0–35.6) | <0.001 | 17.3 (7.8–26.8) | <0.001 |
| **Heart-lung maschine time** | 1 | Reference | | | | | | | |
|  | 2 | 5.9 (-0.5–12.3) | 0.073 | 6.2 (-0.3–12.7) | 0.060 | 5.9 (-0.7–12.5) | 0.082 | 4.0 (-2.3–10.2) | 0.218 |
|  | 3 | 17.4 (10.9–23.8) | <0.001 | 18.1 (11.3–24.9) | <0.001 | 18.1 (11.1–25.2) | <0.001 | 11.5 (4.7–18.3) | 0.001 |
| **Off-pump time** | 1 | Reference | | | | | | | |
|  | 2 | 0.2 (-0.7–1.1) | 0.620 | 0.3 (-0.7–1.2) | 0.584 | 0.3 (-0.6–1.2) | 0.487 | 0.3 (-0.6–1.2) | 0.471 |
|  | 3 | 1.7 (0.8–2.6) | <0.001 | 1.8 (0.8–2.7) | <0.001 | 1.9 (1.0–2.9) | <0.001 | 1.8 (0.9–2.8) | <0.001 |
| **Ventilation time** | 1 | Reference | | | | | | | |
|  | 2 | 8.1 (–6.5–22.6) | 0.276 | 5.6 (-9.1–20.2) | 0.457 | 3.2 (-11.1–17.5) | 0.659 | 1.0 (-13.1–15.2) | 0.889 |
|  | 3 | 43.5 (29.0–58.1) | <0.001 | 38.7 (23.4–54.0) | <0.001 | 30.7 (15.5–45.9) | <0.001 | 23.2 (7.9-38.4) | 0.003 |
| **Length of ICU stay** | 1 | Reference | | | | | | | |
|  | 2 | 0.5 (-0.2–1.2) | 0.187 | 0.3 (-0.4–1.1) | 0.350 | 0.2 (-0.4–0.9) | 0.472 | 0.2 (-0.5–0.8) | 0.650 |
|  | 3 | 2.2 (1.5–2.9) | <0.001 | 2.0 (1.2–2.7) | <0.001 | 1.6 (0.9–2.3) | <0.001 | 1.3 (0.5–2.0) | 0.001 |
| **Length of in-hospital stay** | 1 | Reference | | | | | | | |
|  | 2 | 1.3 (0.1–2.5) | 0.043 | 0.9 (-0.3–2.1) | 0.153 | 0.8 (-0.4–2.0) | 0.193 | 0.7 (-0.5–1.9) | 0.275 |
|  | 3 | 4.1 (2.9–5.3) | <0.001 | 3.3 (2.0–4.6) | <0.001 | 2.8 (1.5–4.1) | <0.001 | 2.2 (0.9–3.6) | 0.001 |

FGF23 tertile 1: ≤50.6 pg/ml, tertile 2: 50.7-89.9 pg/ml, tertile 3: ≥90 pg/ml

* adjusted for age and sex

^§^ adjusted for age, sex, mean arterial blood pressure, sinus rhythm, coronary artery disease, chronic heart failure and smoking status

^#^ adjusted for age, sex, mean arterial blood pressure, sinus rhythm, coronary artery disease, chronic heart failure, smoking status and EuroSCORE
